# Factors associated with the avoidance of glaucoma surgery in secondary glaucoma due to ocular inflammatory disease

**DOI:** 10.1186/s12348-025-00551-0

**Published:** 2025-11-25

**Authors:** Yusuke Nishio, Mutsuhiro Tauchi, Ryota Suga, Naka Shiratori, Kenji Nakamoto, Fumiki Okamoto, Junko Hori

**Affiliations:** 1 Department of Ophthalmology, Nippon Medical School Tama- Nagayama Hospital, 1-7-1 Nagayama, Tama, Tokyo 206-8512 Japan; 2 Department of Ophthalmology, Nippon Medical School, 1-1-5, Sendagi, Bunkyo, Tokyo, 113-8603 Japan

**Keywords:** Glaucoma, Uveitis, Biologics, Steroid, Trabeculectomy

## Abstract

**Background:**

Many cases of hypertensive uveitis progress to visual field narrowing due to difficulty controlling intraocular pressure. The purpose of this retrospective study was to identify factors related to avoidance of trabeculectomy (TLE) in patients with hypertensive uveitis.

**Methods:**

Participants comprised patients diagnosed with ocular inflammatory disease at Nippon Medical School Tama Nagayama Hospital, Ocular Inflammation Service between April 2018 and September 2022. Logistic regression analysis was performed using avoidance of TLE as the objective variable and the following as explanatory variables: age, sex, use of biologics, granulomatous type, background disease, and treatment.

**Results:**

A total of 745 eyes in 480 patients were investigated. Mean age was 57.1 ± 19.6 years, with a male-to-female ratio of 205:275. Hypertensive uveitis was identified in 200 patients (41.7%). The most common causative disease was scleritis (26.5%), followed by sarcoidosis (16.7%), Vogt–Koyanagi–Harada disease (6.0%), Behcet’s disease (5.2%), herpetic keratouveitis (5.0%), HLA-B27-associated uveitis (2.3%), cytomegalovirus iritis/corneal endotheliitis (1.7%), and Posner–Schlossman syndrome (0.6%). The disease type for the remaining 21.9% was unclassifiable. Anti-inflammatory treatments were: topical steroid (100%), sub-conjunctival or sub-Tenon injection of steroid (16.8%), oral steroid (16.6%), oral immunosuppressants (9.1%), and biologics (8.2%). Sixty-eight eyes (9.1%) had a history of TLE. Factors associated with avoidance of TLE were use of biologics (odds ratio [OR] 2.93, *p* = 0.01) and use of oral immunosuppressants (OR 1.42, *p* = 0.08).

**Conclusion:**

Around 9.1% of hypertensive uveitis cases progress to needing TLE. Use of biologics and oral immunosuppressants may help avoid TLE among patients with hypertensive uveitis.

## Background

Ocular inflammatory diseases account for approximately 10% of cases of acquired blindness in individuals over 40 years old in developed countries and are estimated to affect more than 2 million people worldwide [1]. In recent years, significant advances in the diagnosis and treatment of ocular inflammatory diseases have led to an increase in cases where blindness caused by the inflammation itself can be avoided. However, poor control of intraocular pressure (IOP) frequently results in declining visual function, and a correlation between elevated IOP and vision loss has been reported in secondary glaucoma associated with ocular inflammatory diseases (uveitic glaucoma [UG]) [2]. The importance of appropriate IOP management in UG is thus gaining recognition.

The incidence of UG associated with ocular inflammatory diseases varies depending on the definition. When UG is defined solely by elevated IOP, the reported incidence is 17–35.4% [3]. When defined as elevated IOP with the use of IOP-lowering drugs, the incidence is 18–27% [4], and when defined as elevated IOP with glaucomatous visual field defects, the incidence is reported as 7–10% [5]. One retrospective study based on a database of insurance claims in the United States reported that 20% of patients with non-infectious uveitis developed UG within a 5-year period [6]. Further, a multicentre joint study investigating the incidence of UG with non-infectious uveitis found that one in three patients exhibited an IOP ≥21 mmHg, and one in seven patients had an IOP ≥30 mmHg at two years after the onset of ocular inflammation [7]. Eyes also seem to be polarised into those that are prone to increased IOP and those that are not [7], and once elevated IOP arises, increases continue for over 10 years [2]. In addition, epidemiological studies have revealed that the risk of developing UG is particularly high in cases of anterior uveitis, chronic uveitis, and in elderly patients [8]. Since UG often results in significant elevations and large fluctuations in IOP, the average time from IOP elevation to the development of glaucomatous visual field defects is reportedly 1.2 years, shorter than that in primary open-angle glaucoma (POAG) [9]. Moreover, an analysis of big data in the United Kingdom showed that the progression rate of visual field loss in UG is 1.9 times faster than that in POAG [10].

The approach to UG involves coordinated, stepwise treatment by both ocular inflammation clinics and glaucoma clinics. In the initial stage, where active inflammation and elevated IOP are present but the underlying disease has not yet been identified, the ocular inflammation clinic conducts systemic investigations to determine the underlying pathology. Next, in the stage where the disease has been identified but both inflammation and IOP remain poorly controlled, anti-inflammatory treatments targeting the underlying disease and medication-based IOP-lowering therapies are administered. Finally, when inflammation is in remission but IOP control remains inadequate, glaucoma surgery is considered while continuing anti-inflammatory treatment for the underlying disease. At this stage, IOP becomes the primary criterion for deciding whether glaucoma surgery should be performed for UG. Generally, UG that requires glaucoma surgery exhibits significant IOP elevation, and in some cases, the outflow pathway of aqueous humour may be obstructed by peripheral anterior synechiae (PAS). In such situations, filtration surgeries, including trabeculectomy (TLE), are considered the most effective treatment. Ideally, TLE should be performed during a period of sustained remission achieved through sufficient anti-inflammatory treatment. However, in some cases, surgery must be performed even when remission has not been achieved, to preserve visual function [11]. In cases where the IOP elevation is mild or where steroid-induced glaucoma is strongly suspected, a less invasive TLE may be performed. However, as mentioned earlier, UG often presents with severe visual field defects and significantly elevated IOP, making filtration surgery the preferred choice for initial glaucoma surgery in many cases. Among the filtration surgeries, TLE is widely performed for UG worldwide [12].

However, TLE for UG has long been recognised as having poor surgical outcomes [13]. While the success rate of TLE for UG has reportedly improved to 51–90% with the use of mitomycin C, outcomes remain mixed. Some studies have suggested no significant difference in success rates between UG and POAG for initial TLE, while others have indicated inferior outcomes for UG compared to POAG [11]. In Japan, one study reported that the rate of controlling IOP to < 21 mmHg for 3 years was 71.3% for UG, compared to 89.7% for POAG [14].

TLE carries certain risks, including haemorrhagic complications, induced astigmatism, and bleb-related ocular infection [15]. Particularly in UG, concerns regarding postoperative hypotony and progression of cataracts are even greater than in POAG, making the surgical invasiveness a non-negligible issue [16]. On the other hand, recent years have seen a paradigm shift in the treatment of non-infectious uveitis with the advent of biologics and immunosuppressants, which have become mainstream in the management of ocular inflammation [17]. With these potent immunosuppressive therapies, TLE might be avoided by reducing or discontinuing steroids and enhancing anti-inflammatory measures. However, no previous studies appear to have examined the relationship between immunosuppressive therapy and glaucoma surgery. The present study therefore investigated the potential usefulness of appropriate immunosuppressive therapy in avoiding TLE in hypertensive uveitis.

## Materials and methods

A retrospective review was conducted for the medical records of 480 patients (745 eyes) diagnosed with ocular inflammatory diseases at the Ocular Inflammation Service of Nippon Medical School Tama Nagayama Hospital between April 2018 and September 2022. The study aimed to identify factors associated with avoidance of TLE in cases of hypertensive uveitis. Diagnoses were made based on ocular findings, clinical test results such as fluorescein fundus angiography, serological tests, and X-ray examinations. Cases for which a definitive diagnosis could not be established were considered “unclassifiable.”

In this study, hypertensive uveitis was defined for cases in which IOP was > 21 mmHg on three or more occasions, regardless of the presence of glaucomatous optic neuropathy (GON), while using glaucoma eye drops. The anatomical location of ocular inflammation was classified into anterior uveitis, intermediate uveitis, posterior uveitis, and panuveitis. Cases involving inflammation across two anatomical regions were categorised based on the predominant area of inflammation, while those extending from the anterior to posterior segments were classified as panuveitis. Although various surgical techniques for glaucoma are currently available in Japan, the decision to perform surgery and the choice of surgical method in this study were made on a case-by-case basis by an ocular inflammation specialist (J.H.) and a glaucoma specialist (Y.N.). The indication for glaucoma surgery was defined as uncontrolled IOP despite medical therapy, or repeated episodes of acute IOP elevation with rapid visual field progression. During the study period, TLE was the only glaucoma surgery performed for hypertensive uveitis.

The primary outcome measures were analysed using logistic regression with the forced entry method, using avoidance of TLE and presence of hypertensive uveitis as the dependent variables. Explanatory variables included age, sex, use of biologics, granulomatous type, presence of background diseases, use of topical immunosuppressants, use of oral immunosuppressants, use of oral steroids, subconjunctival injection of triamcinolone acetonide (SCTA), and sub-Tenon injection of triamcinolone acetonide (STTA). Variables showing values of *p* < 0.25 in univariate analyses were included in the logistic regression model. Secondary outcome measures included the incidence of hypertensive uveitis and the rate of TLE among different ocular inflammatory diseases, as well as the incidence of hypertensive uveitis and the rate of TLE based on the anatomical location of inflammation. Factors were analysed using cross-tabulation and Pearson’s chi-square test. To assess multicollinearity in the logistic regression analysis, the variance inflation factor was calculated. The goodness of fit of the model was evaluated using the Hosmer–Lemeshow test.

This study was conducted in compliance with the Declaration of Helsinki and the Ethical Guidelines for Medical and Health Research Involving Human Subjects. It was approved by the Ethics Committee of Nippon Medical School Tama Nagayama Hospital (Approval Number: F-2022-041). A two-sided significance level of 5% was used. Data analysis was performed using SPSS Statistics Ver. 27 for Windows (IBM, USA).

## Results

**Fig. 1:**
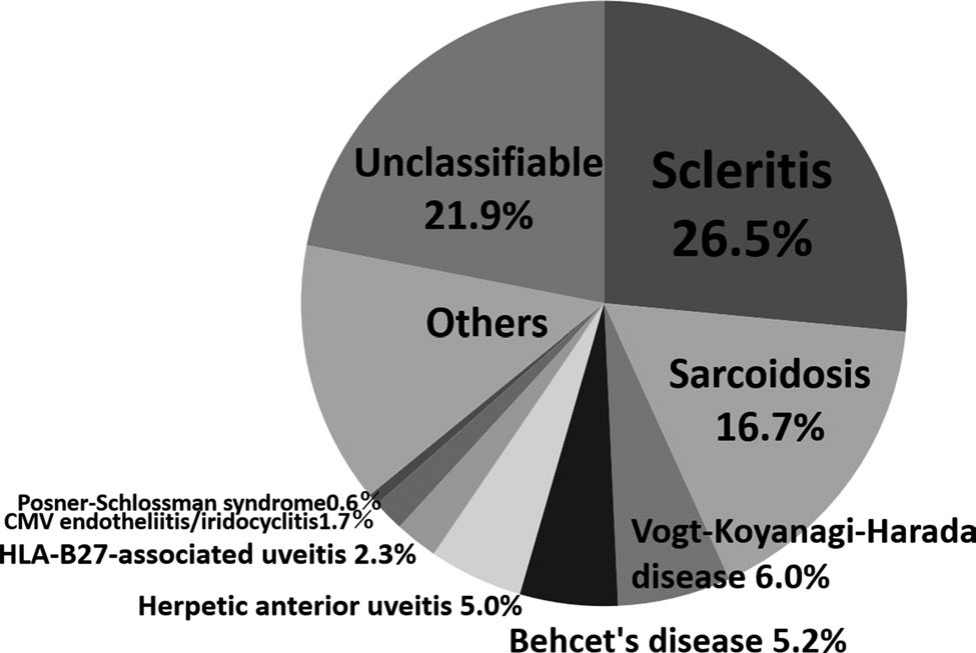
Frequency of ocular inflammatory diseases. In this study, the most frequent disease was scleritis, at 26.5%

Mean (± standard deviation) age at the initial visit was 57.1 ± 19.6 years, and mean disease duration was 3.68 ± 6.11 years (median, 2.00 years). Categorised by symptoms of ocular inflammation, granulomatous uveitis was the most common (316 eyes, 42.4%), followed by non-granulomatous uveitis (242 eyes, 32.5%) and scleritis (187 eyes, 25.1%). Classified by the anatomical location of inflammation, panuveitis was the most frequent, occurring in 33.6% of cases. This was followed by anterior uveitis in 211 eyes (28.3%), scleritis in 187 eyes (25.1%), posterior uveitis in 55 eyes (7.4%), and intermediate uveitis in 42 eyes (5.6%). Underlying systemic autoimmune diseases associated with ocular inflammatory were present in 110 eyes (14.5%) (Table 1). Disease classification was possible in 375 cases (78.1%). Among these, episcleritis/scleritis was the most common, occurring in 127 cases (26.5%), followed by sarcoidosis in 80 cases (16.7%), Vogt–Koyanagi–Harada disease in 29 cases (6.0%), Behcet’s disease in 25 cases (5.2%), herpetic keratouveitis in 24 cases (5.0%), HLA-B27-associated uveitis in 11 cases (2.3%), cytomegalovirus (CMV) endotheliitis/iridocyclitis in 8 cases (1.7%), and Posner–Schlossman syndrome in 3 cases (0.6%) (Fig. 1). Of note here is that our specialised outpatient clinic for ocular inflammation focuses on refractory scleritis, and we thus receive many referrals for scleritis from all over Japan, which accounts for the breakdown of the above-mentioned diseases.

**Table 1:** Patient background

**Type of inflammation**	**Eyes**	**Site of inflammation**	**Eyes**
Granulomatous uveitis	316	Anterior uveitis	211
Non-granulomatous uveitis	242	Intermediate uveitis	42
Scleritis	187	Posterior uveitis	55
		Panuveitis	250
Background disease	110	Scleritis	187
**Oral immunosuppressants**	**Eyes**	**Biologics**	**Eyes**
Cyclosporine	61	Adalimumab	44
Methotrexate	4	Infliximab	12
Azathioprine	3	Golimumab	2
		Etanercept	2
SCTA	15	Tocilizumab	2
STTA	110	Sarilumab	2

This table shows the type and site of inflammation in 745 eyes of 480 patients. Oral immunosuppressants were administered to 68 eyes, biologics to 61 eyes, oral steroids to 124 eyes, and topical immunosuppressants to 106 eyes. Background disease refers to systemic autoimmune diseases associated with ocular inflammatory diseases

Treatment was oral steroids in 124 eyes (16.6%), and oral immunosuppressants other than steroids in 68 eyes (9.1%). The breakdown of the latter included cyclosporine as the most common, in 61 eyes (8.2%), followed by methotrexate in 4 eyes (0.5%) and azathioprine in 3 eyes (0.4%). In addition, 61 eyes (8.2%) were treated with biologics. The breakdown of these agents included adalimumab (ADA) as the most frequently used, in 44 eyes (5.9%), followed by infliximab (IFX) in 12 eyes (1.6%), and golimumab, etanercept, tocilizumab, and sarilumab in 2 eyes (0.3%) each. All these prescriptions included those from the Department of Rheumatology for underlying systemic diseases. For local ocular immunosuppressive therapy, SCTA was administered in 15 eyes (2.0%), and STTA in 110 eyes (14.8%) (Table 1).

At the initial visit, 81 eyes had a history of cataract surgery. During the study period, cataract surgery was performed on 72 eyes, laser peripheral iridotomy on 5 eyes, and TLE on 68 eyes. Among all subjects, 200 cases (41.7%) were diagnosed with hypertensive uveitis, and TLE was performed in 36 cases (7.5%). In cases that required TLE, the mean preoperative glaucoma medication score was 5.9 ± 1.3, and the mean IOP was 23.8 ± 7.4 mmHg. The medication score was calculated by assigning one point for each single topical agent, two points for each fixed combination, and one point for each oral medication. The incidence of hypertensive uveitis by ocular inflammatory disease type is shown in Table 2, and TLE rates are shown in Table 3. The incidence of hypertensive uveitis was highest in Posner–Schlossman syndrome (100%), followed by Vogt–Koyanagi–Harada disease and CMV endotheliitis/iridocyclitis, with both exceeding 60%. On the other hand, TLE rate was highest in CMV endotheliitis/iridocyclitis, followed by Vogt–Koyanagi–Harada disease and Behcet’s disease. Mean deviation (MD) values for the Humphrey visual field were − 6.09 ± 9.22 dB in the hypertensive uveitis group and − 13.03 ± 12.90 dB in the TLE group. Visual field index was 63.64 ± 42.15% in the hypertensive uveitis group and 58.57 ± 36.50% in the TLE group.

**Table 2:** Hypertensive uveitis incidence by ocular inflammatory disease (%)

Diseases	Rate (cases)	Rate (eyes)
Posner–Schlossman syndrome	100.0 (3/3)	100.0 (3/3)
Vogt–Koyanagi–Harada disease	62.1 (18/29)	60.4 (32/53)
CMV endotheliitis/iridocyclitis	62.5 (5/8)	60.0 (6/10)
Scleritis, episcleritis	44.1 (56/127)	47.6 (89/187)
Sarcoidosis	46.3 (37/80)	46.9 (69/147)
Behcet’s disease	36.0 (9/25)	42.2 (19/45)
Unclassifiable	32.4 (34/105)	39.9 (57/143)
HLA-B27-associated uveitis	36.4 (4/11)	35.3 (6/17)
Herpetic anterior uveitis	8.3 (2/24)	16.0 (4/25)
Others	47.1 (32/68)	39.1 (45/115)
Total	**41.7** (200/480)	**44.3** (330/745)

This table shows the incidence of hypertensive uveitis by underlying ocular inflammatory disease. Of the 480 cases, 200 cases (41.7%) developed hypertensive uveitis and of the 745 eyes, 330 eyes (44.3%) developed hypertensive uveitis

**Table 3:** Rate of TLE by ocular inflammatory disease (%)

Diseases	Rate (cases)	Rate (eyes)
CMV endotheliitis/iridocyclitis	25.0 (2/8)	30.0 (3/10)
Vogt–Koyanagi–Harada disease	20.7 (6/29)	17.0 (9/53)
Behcet’s disease	12.0 (3/25)	11.1 (5/45)
Scleritis, episcleritis	5.5 (7/127)	8.0 (15/187)
Sarcoidosis	8.8 (7/80)	7.5 (11/147)
HLA-B27-associated uveitis	9.1 (1/11)	5.9 (1/17)
Unclassifiable	3.8 (4/105)	5.6 (8/143)
Posner–Schlossman syndrome	0.0 (0/3)	0.0 (0/3)
Herpetic anterior uveitis	0.0 (0/24)	0.0 (0/25)
Others	8.8 (6/68)	13.9 (16/115)
Total	**7.5** (36/480)	**9.1** (68/745)

This table shows the rate of TLE by ocular inflammatory diseases. TLE was performed in 36 of 480 cases (7.5%) and in 68 of 745 eyes (9.1%)

**Fig. 2:**
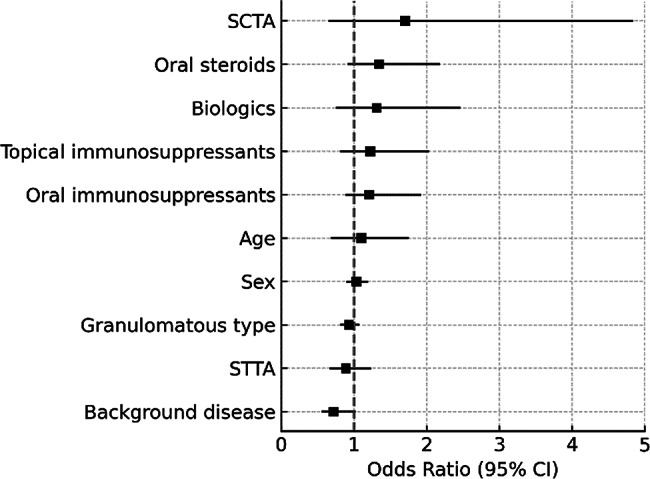
Factors associated with development of uveitic hypertensive uveitis. Logistic regression analysis was conducted to identify factors associated with the development of hypertensive uveitis, but no significant factors were found. The Hosmer-Lemeshow test indicated good model fit (*p* = 0.063) and the model demonstrated high discriminant accuracy (86.3%)

**Fig. 3:**
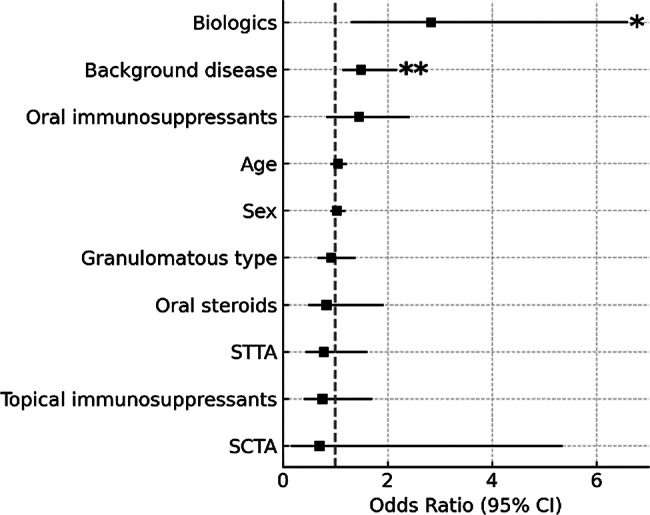
Factors associated with avoidance of TLE. Logistic regression analysis was conducted to identify factors associated with avoiding TLE. Use of biological agents (OR 2.93, 95%CI 1.30–6.60; *, *p* = 0.01) and the presence of underlying systemic disease (OR 1.45, 95%CI 1.12–2.09; **, *p* = 0.008) were identified as significant factors. The Hosmer-Lemeshow test indicated good model fit (*p* = 0.64), and the model demonstrated high discriminant accuracy (91.1%)

Logistic regression analysis was conducted to identify factors associated with the development of hypertensive uveitis, but no significant factors were found. The only factors found to be significantly associated with avoiding TLE were the use of biologics (odds ratio [OR] 2.93, 95% confidence interval [CI] 1.30–6.60; *p* = 0.01) and the presence of underlying systemic disease (OR 1.45, 95%CI 1.12–2.09; *p* = 0.008). The Hosmer–Lemeshow test indicated good model fit (*p* = 0.64), and the model demonstrated high discriminant accuracy of 91.1% (Figs. 2 and 3).

Table 4 shows the incidence of hypertensive uveitis and rates of TLE according to the area of inflammation. A cross-tabulation of inflamed areas with the presence or absence of hypertensive uveitis and TLE was created, and chi-square tests were conducted to calculate adjusted residuals. The incidence of hypertensive uveitis was relatively high in scleritis and relatively low in posterior uveitis. On the other hand, no significant difference in TLE rates was seen among different areas of inflammation.

**Table 4:** Hypertensive uveitis incidence and TLE execution rate by inflammation site

	**Anterior**	**Intermediate**	**Posterior**	**Pan**	**Scleritis**
hypertensive uveitis (-)	132	10	39	134	100
hypertensive uveitis (+) (adjusted residual)	87 (-1.6)	3 (-1.6)	17 (-2.2*)	121 (1.3)	102 (2.1*)
hypertensive uveitis (%)	39.7%	23.1%	30.4%	47.5%	50.5%
TLE (-)	200	11	52	229	185
TLE (+) (adjusted residual)	19 (-0.3)	2 (0.8)	4 (-0.5)	26 (0.7)	17 (-0.4)
TLE (%)	8.7%	15.4%	7.1%	10.2%	8.4%
		**Pearson’s chi-square test**		**Coefficient of association**	
hypertensive uveitis		*p* = 0.012		φ = 0.131 (*p* = 0.012)	
TLE		*p* = 0.843		φ = 0.043 (*p* = 0.843)	

Cross-tabulation was created to examine the relationships between the incidence of hypertensive uveitis and inflammation site as well as the rate of TLE and inflammation site. Chi-square tests were performed and adjusted residuals were calculated (*). The incidence of hypertensive uveitis among sites of ocular inflammation was lower in posterior uveitis and higher in scleritis. On the other hand, no difference was seen in TLE execution rates

Logistic regression analysis revealed that the group with underlying systemic disease was able to largely avoid TLE. Consequently, cross-tabulation was performed to examine the relationship between the presence of underlying diseases and the use of biologics. Chi-square tests were conducted, and adjusted residuals were calculated. The usage rate of biologics was significantly higher in the group with underlying disease (14.5%) compared to the group without (7.1%) (Table 5).

**Table 5:** Use of biologics by presence of background disease

	Background disease (-)	Background disease (+)	Total
Biologics (-) (adjusted residual)	590 (2.6*)	94 (-2.6*)	684
Biologics (+) (adjusted residual)	45 (-2.6*)	16 (2.6*)	61
Biologics usage rate	7.1%	14.5%	8.2%
Total	635	110	745

Pearson’s chi-square test *p* = 0.008

Coefficient of association φ = 0.097 (*p* = 0.008)

Cross-tabulation was created to examine the relationship between the presence of underlying disease and the use of biologics. Chi-square tests were performed and adjusted residuals were calculated (*). The use of biologics was significantly higher in the group with underlying diseases (14.5%) compared to the group without (7.1%)

## Discussion

In this study, hypertensive uveitis was observed in over 40% of cases with ocular inflammatory disease, higher than the previously reported rates of 28.6–35.0% [18]. This may be attributed to the higher number of cases with Vogt–Koyanagi–Harada disease, CMV endotheliitis/iridocyclitis, and severe scleritis, which are associated with higher incidences of hypertensive uveitis. In addition, as a specialised ocular inflammation clinic in a university hospital, more severe cases were likely referred to this centre. The usage rate of biologics in patients with ocular inflammation was 8.2%, exceeding the previously reported rate of 1.2%. Among the biologics used, the tumour necrosis factor (TNF)-α inhibitor ADA was the most frequently prescribed, with scleritis being the most common condition treated. The efficacy of TNF-α inhibitors in non-infectious uveitis and scleritis has been demonstrated [19], which likely explains these results.

In hypertensive uveitis, four mechanisms of IOP elevation are recognised: (i) trabeculitis type, where IOP increases in association with inflammation; (ii) steroid-induced type, where IOP gradually increases due to the use of steroids; (iii) acute IOP elevation type, characterised by a sudden increase in IOP with inflammation, often due to angle closure, such as in iris bombe; and (iv) chronic IOP elevation type, where IOP gradually increases due to increased outflow resistance over time [20]. These four mechanisms interact in a multi-mechanistic manner. The trabeculitis type is expected to show reduced IOP with anti-inflammatory treatment, but if the reduction is insufficient, TLE may be required [21]. The steroid-induced type involves attempting to taper the usage of steroids, where feasible, and reconstructive surgery for the outflow pathway may be effective [22]. The acute IOP elevation type requires sufficient anti-inflammatory treatment, followed by consideration of laser peripheral iridotomy or cataract surgery [23]. Regarding the chronic IOP elevation type, IOP reduction cannot be expected through glaucoma drug treatment alone, and filtration surgery such as TLE is often required [24]. As hypertensive uveitis treatment strategies differ based on the mechanism causing elevated IOP, tailoring the glaucoma therapy to the specific underlying conditions is crucial.

However, 28.6–61.1% of UG cases are reportedly associated with steroid-induced glaucoma, indicating that the frequency of this state is by no means negligible [25]. The proportion of steroid responders is estimated to be around 5% in normal eyes, but has been reported to exceed 90% in POAG [26]. Cases of UG often show little excess outflow resistance in the main pathway [27], making the case more susceptible to IOP elevation due to steroids. The risk of steroid-induced IOP elevation also varies depending on the route and frequency of administration. According to previous reports, the risk of IOP elevation doubles with oral prednisolone doses ≥7.5 mg and with the use of 0.1% betamethasone eye drops more than twice a day [28]. Therefore, in the treatment of glaucoma in hypertensive uveitis, clinicians always need to be aware of the possibility of steroid-induced glaucoma.

In Japan, both ADA and IFX are covered by the national health insurance system for the treatment of non-infectious uveitis. ADA is covered by insurance for refractory non-infectious intermediate, posterior, and panuveitis when conventional treatments are insufficient, while IFX is only covered for uveitis associated with Behcet’s disease. For the systemic treatment of refractory non-infectious uveitis, biologics such as ADA are often used after administering oral steroids to enhance the anti-inflammatory effects and facilitate the tapering or discontinuation of steroids [29]. ADA has been extensively studied in various clinical trials, including the VISUAL trials, for its efficacy in non-infectious uveitis [30]. While outcomes have varied across reports, combining ADA with treatment for non-infectious uveitis has been demonstrated to reduce the daily dosage of oral prednisolone by approximately 7–10 mg and to enable discontinuation of oral steroids in about 26–65% of cases [31].

The present study investigated factors associated with avoidance of TLE in hypertensive uveitis, revealing the use of biologics and the presence of underlying disease were identified as significant factors. Reports analysing cytokine concentrations in the aqueous humour of UG patients have confirmed elevations in interleukin (IL)-6, IL-8, TNF-α, vascular endothelial growth factor (VEGF), and monocyte chemotactic protein-1 (MCP-1) [32]. IL-6 and IL-8 are considered factors associated with the induction of oxidative stress, leading to trabecular meshwork cell death, and are also reported to influence the differentiation and proliferation of fibroblasts, along with TNF-α [33]. VEGF, while widely known as a growth factor associated with the vascular endothelium, also promotes the migration of inflammatory cells such as macrophages and neutrophils and stimulates the differentiation and proliferation of fibroblasts [34]. MCP-1 is a chemokine that facilitates the migration of monocytes and macrophages, thereby recruiting more inflammatory cells [35]. To the best of our knowledge, no studies have compared cytokine concentrations in the aqueous humour before and after administration of biologics in hypertensive uveitis. However, potent immunosuppressive treatment is hypothesised to be achieved with biologics and may have reduced the above-mentioned cytokine concentrations in the anterior chamber, suppressed inflammation and fibrosis of the trabecular meshwork, preserved its function, and ultimately decreased the frequency of TLE. Further, high efficacy of anti-inflammatory and steroid-sparing effects can be achieved with the use of biologics, and combined with the ability to prevent severe trabeculitis-induced elevation of IOP, may have contributed to the avoidance of TLE.

This study has several limitations. First, due to the characteristics of our department, which frequently receives referrals for severe scleritis, the incidence of hypertensive uveitis by anatomical location showed a predominance of scleritis. This suggests potential bias in the types of ocular inflammatory diseases associated with hypertensive uveitis included in this investigation. In addition, the severity of inflammatory activity was not considered, and the study was a single-centre, retrospective study with a limited sample size and observation period. Further, the study did not investigate the presence of comorbidities with other types of glaucoma, such as POAG. Moreover, as the study aimed to examine the impact of appropriate immunosuppressive treatment on TLE avoidance in hypertensive uveitis, factors such as IOP, gonioscopic findings, mechanisms of IOP elevation, and family history of glaucoma were not considered. In addition, because the treatment protocols for biologic agents and systemic immunosuppressive therapies were heterogeneous, statistically evaluating their effects on IOP was difficult in this study. Because this was a retrospective study, gonioscopic findings, mechanisms of IOP elevation, and severity of inflammation varied over time, and due to limitations in data extraction, these factors could not be incorporated into the analysis. In addition, since only 68 cases underwent TLE, adding too many explanatory variables relative to the number of events could have reduced the statistical power of the logistic regression analysis. Therefore, from the perspective of events per variable (EPV), explanatory variables were mainly limited to treatment-related factors. Future studies should incorporate these factors for a more comprehensive analysis. Further investigations that take these additional factors into account are warranted.

## Conclusions

This study demonstrated that hypertensive uveitis is less likely to develop in cases of posterior uveitis and more likely in cases of scleritis. However, no significant differences in the rate of TLE were observed across different anatomical areas of ocular inflammation. The use of biologics and oral immunosuppressants also appeared effective in avoiding TLE in hypertensive uveitis. These findings suggest that in cases of hypertensive uveitis where concurrent pharmacotherapies make controlling IOP difficult, reducing the use of steroids and actively employing biologics and immunosuppressants may be useful for avoiding TLE.

## Data Availability

The datasets used during the current study are available from the corresponding author on reasonable request.

## Abbreviations

ADA: Adalimumab
CI: Confidence interval
CMV: Cytomegalovirus
GON: Glaucomatous optic neuropathy
HLA: HUMAN leukocyte antigen
IFX: Infliximab
IL: Interleukin
IOP: Intraocular pressure
MCP-1: Monocyte chemotactic protein-1
MD: Mean deviation
OR: Odds ratio
PAS: Peripheral anterior synechia
POAG: Primary open-angle glaucoma
SCTA: Subconjunctival triamcinolone acetonide
STTA: Sub-Tenon triamcinolone acetonide injection
TLE: Trabeculectomy
TNF-α: Tumour necrosis factor-α
UG: Uveitic glaucoma
VEGF: Vascular endothelial growth factor

## References

[1] Siddique SS, Suelves AM, Baheti U, Foster CS (2013 Jan-Feb) Glaucoma and uveitis. Surv Ophthalmol. 58(1):1–10. 10.1016/j.survophthal.2012.04.006 10.1016/j.survophthal.2012.04.006 23217584

[2] Neri P, Azuara-Blanco A, Forrester JV (2004) Incidence of glaucoma in patients with uveitis. J Glaucoma 13(6):461–465. 10.1097/01.ijg.0000146391.77618.d0 15534470 10.1097/01.ijg.0000146391.77618.d0

[3] Merayo-Lloves J, Power WJ, Rodriguez A, Pedroza-Seres M, Foster CS (1999) Secondary glaucoma in patients with uveitis. Ophthalmologica 213(5):300–304. 10.1159/000027443 10516518 10.1159/000027443

[4] Takahashi T, Ohtani S, Miyata K, Miyata N, Shirato S, Mochizuki M (2002 Sep-Oct) A clinical evaluation of uveitis-associated secondary glaucoma. Jpn J Ophthalmol 46(5):556–562. 10.1016/s0021-5155(02)00549-x 10.1016/s0021-5155(02)00549-x 12457916

[5] Heinz C, Koch JM, Zurek-Imhoff B, Heiligenhaus A (2009) Prevalence of uveitic secondary glaucoma and success of nonsurgical treatment in adults and children in a tertiary referral center. Ocul Immunol Inflamm 17(4):243–248. 10.1080/09273940902913035 10.1080/09273940902913035 19657977

[6] Dick AD, Tundia N, Sorg R, Zhao C, Chao J, Joshi A, Skup M (2016) Risk of Ocular Complications in Patients with Noninfectious Intermediate Uveitis, Posterior Uveitis, or Panuveitis. Ophthalmology. ;123(3):655 – 62. 10.1016/j.ophtha.2015.10.028. Epub 2015 Dec 19. Erratum in: Ophthalmology. 2016;123(11):2439. 10.1016/j.ophtha.2016.09.006. 10.1016/j.ophtha.2015.10.028 26712559

[7] Daniel E, Pistilli M, Kothari S, Khachatryan N, Kaçmaz RO, Gangaputra SS, Sen HN, Suhler EB, Thorne JE, Foster CS, Jabs DA, Nussenblatt RB, Rosenbaum JT, Levy-Clarke GA, Bhatt NP, Kempen JH, Systemic Immunosuppressive Therapy for Eye Diseases Research Group (2017) Risk of ocular hypertension in adults with noninfectious uveitis. Ophthalmology 124(8):1196–1208. 10.1016/j.ophtha.2017.03.041Epub 2017 Apr 19 28433444 10.1016/j.ophtha.2017.03.041 PMC5522760

[8] Niederer RL, Wong ABC, Ma T, Chew S, Sims J (2023) Predictors of glaucoma in patients with uveitis and scleritis. Eye (Lond) 37(6):1254–1257. 10.1038/s41433-022-02101-7Epub 2022 May 24 35610358 10.1038/s41433-022-02101-7 PMC10101954

[9] Ma T, Sims JL, Bennett S, Chew S, Niederer RL (2022) High rate of conversion from ocular hypertension to glaucoma in subjects with uveitis. Br J Ophthalmol 106(11):1520–1523. 10.1136/bjophthalmol-2021-318809Epub 2021 May 21 34020941 10.1136/bjophthalmol-2021-318809

[10] Liu X, Kelly SR, Montesano G, Bryan SR, Barry RJ, Keane PA, Denniston AK, Crabb DP (2019) Evaluating the impact of uveitis on visual field progression using Large-Scale Real-World data. Am J Ophthalmol 207:144–150 Epub 2019 Jun 26 31251907 10.1016/j.ajo.2019.06.004

[11] Carreño E, Villarón S, Portero A, Herreras JM, Maquet JA, Calonge M (2011) Surgical outcomes of uveitic glaucoma. J Ophthalmic Inflamm Infect 1(2):43–53. 10.1007/s12348-010-0012-8Epub 2010 Nov 18 21484172 10.1007/s12348-010-0012-8 PMC3102843

[12] Halkiadakis I, Konstantopoulou K, Tzimis V, Papadopoulos N, Chatzistefanou K, Markomichelakis NN (2024) Update on diagnosis and treatment of uveitic glaucoma. J Clin Med 13(5):1185. 10.3390/jcm13051185 38592059 10.3390/jcm13051185 PMC10931771

[13] Almobarak FA, Alharbi AH, Morales J, Aljadaan I (2018) The influence of mitomycin C concentration on the outcome of trabeculectomy in uveitic glaucoma. Int Ophthalmol 38(6):2371–2379. 10.1007/s10792-017-0737-6Epub 2017 Oct 14 29032434 10.1007/s10792-017-0737-6

[14] Iwao K, Inatani M, Seto T, Takihara Y, Ogata-Iwao M, Okinami S, Tanihara H (2014) Long-term outcomes and prognostic factors for trabeculectomy with mitomycin C in eyes with uveitic glaucoma: a retrospective cohort study. J Glaucoma 23(2):88–94. 10.1097/IJG.0b013e3182685167 22895522 10.1097/IJG.0b013e3182685167

[15] Hoffmann EM, Hengerer F, Klabe K, Schargus M, Thieme H, Voykov B (2021) Aktuelle Glaukomchirurgie [Glaucoma surgery today]. Ophthalmologe. ;118(3):239–247. German. 10.1007/s00347-020-01146-x. Erratum in: Ophthalmologe. 2021;118(Suppl 2):183. 10.1007/s00347-021-01457-7. 10.1007/s00347-020-01146-x PMC8219657 32632493

[16] Almobarak FA, Alharbi AH, Aljadaan I, Aldhibi H (2021) Long-term outcomes of initial trabeculectomy in glaucoma associated with granulomatous and non-granulomatous uveitis. Int Ophthalmol 41(10):3459–3470. 10.1007/s10792-021-01910-1Epub 2021 Jun 7 34097195 10.1007/s10792-021-01910-1

[17] Bitossi A, Mattioli I, Bettiol A, Palermo A, Malandrino D, Bacherini D, Virgili G, Giansanti F, Vannozzi L, Silvestri E (2023) Non-anti TNFα biologic agents for noninfectious uveitis associated with systemic inflammatory diseases: a systematic review. Expert Rev Clin Immunol 19(5):549–560 Epub 2023 Mar 27 36939549 10.1080/1744666X.2023.2193687

[18] Sonoda KH, Hasegawa E, Namba K, Okada AA, Ohguro N, Goto H, JOIS (Japanese Ocular Inflammation Society) Uveitis Survey Working Group (2021) Epidemiology of uveitis in japan: a 2016 retrospective nationwide survey. Jpn J Ophthalmol 65(2):184–190. 10.1007/s10384-020-00809-1Epub 2021 Mar 11 33694024 10.1007/s10384-020-00809-1

[19] Chauhan K, Tyagi M (2024) Update on non-infectious uveitis treatment: anti-TNF-alpha and beyond. Front Ophthalmol (Lausanne) 4:1412930. 10.3389/fopht.2024.1412930 39157460 10.3389/fopht.2024.1412930 PMC11327136

[20] Kalogeropoulos D, Sung VC, Curr Glaucoma J Pract (2018) Sep-Dec ;12(3):125–138. 10.5005/jp-journals-10028-1257 10.5005/jp-journals-10028-1257 PMC6647826 31354205

[21] Pillai MR, Balasubramaniam N, Wala N, Mathews AM, Tejeswi B, Krishna H, Ishrath D, Rathinam SR, Sithiq Uduman SM (2024) Glaucoma in uveitic eyes: long-term clinical course and management measures. Ocul Immunol Inflamm 32(6):1041–1047 Epub 2023 May 4 37140329 10.1080/09273948.2023.2202740

[22] Mosaed S (2020) Ab interno trabeculotomy in uveitic glaucoma: confirmation of original results with extended applications. Clin Exp Ophthalmol 48(1):12–13. 10.1111/ceo.13699 32036631 10.1111/ceo.13699

[23] Ikegawa W, Suzuki T, Namiguchi K, Mizoue S, Shiraishi A, Ohashi Y (2016) Changes in anterior segment morphology of Iris Bombe before and after laser peripheral iridotomy in patients with uveitic secondary glaucoma. J Ophthalmol 2016:8496201. 10.1155/2016/8496201 27872755 10.1155/2016/8496201 PMC5107836

[24] Škrlová E, Svozílková P, Heissigerová J, Fichtl M (2023) Pathogenesis and current methods of treatment, of secondary uveitic glaucoma. a review. Cesk Slov Oftalmol 79(3):111–115 English. 10.31348/2023/7 10.31348/2023/7 36858946

[25] Shrestha S, Thapa M, Shah DN (2014) Pattern of intraocular pressure fluctuation in uveitic eyes treated with corticosteroids. Ocul Immunol Inflamm 22(2):110–115. 10.3109/09273948.2013.824106 24131295 10.3109/09273948.2013.824106

[26] Razeghinejad MR, Katz LJ (2012) Steroid-induced iatrogenic glaucoma. Ophthalmic Res 47(2):66–80. 10.1159/000328630 21757964 10.1159/000328630

[27] Alaghband P, Baneke AJ, Galvis E, Madekurozwa M, Chu B, Stanford M, Overby D, Lim KS (2019) Aqueous humor dynamics in uveitic eyes. Am J Ophthalmol 208:347–355 Epub 2019 Aug 30 31473215 10.1016/j.ajo.2019.08.018

[28] Yorio T, Patel GC, Clark AF (2020) Glucocorticoid-induced ocular hypertension: origins and new approaches to minimize. Expert Rev Ophthalmol 15(3):145–157 Epub 2020 May 14 38274668 10.1080/17469899.2020.1762488 PMC10810227

[29] Chang SM, St Peter DM, Im LT, Munir WM, Schocket LS (2022) Dexamethasone implant migration in an eye with congenital glaucoma: a case report and review of the literature. Eur J Ophthalmol 32(5):NP46–NP50. Epub 2021 Mar 29 10.1177/11206721211005696 33781105 10.1177/11206721211005696

[30] Nguyen QD, Merrill PT, Jaffe GJ, Dick AD, Kurup SK, Sheppard J, Schlaen A, Pavesio C, Cimino L, Van Calster J, Camez AA, Kwatra NV, Song AP, Kron M, Tari S, Brézin AP (2016) Adalimumab for prevention of uveitic flare in patients with inactive non-infectious uveitis controlled by corticosteroids (VISUAL II): a multicentre, double-masked, randomised, placebo-controlled phase 3 trial. Lancet. ;388(10050):1183-92. 10.1016/S0140-6736(16)31339-3. Epub 2016 Aug 16. Erratum in: Lancet. 2016;388(10050):1160. doi: 10.1016/S0140-6736(16)31538-0 10.1016/S0140-6736(16)31339-3 27542302

[31] Suhler EB, Adán A, Brézin AP, Fortin E, Goto H, Jaffe GJ, Kaburaki T, Kramer M, Lim LL, Muccioli C, Nguyen QD, Van Calster J, Cimino L, Kron M, Song AP, Liu J, Pathai S, Camez A, Schlaen A, van Velthoven MEJ, Vitale AT, Zierhut M, Tari S, Dick AD (2018) Safety and efficacy of adalimumab in patients with noninfectious uveitis in an ongoing Open-Label study: VISUAL III. Ophthalmology 125(7):1075–1087. 10.1016/j.ophtha.2017.12.039Epub 2018 Feb 9 29429764 10.1016/j.ophtha.2017.12.039

[32] Inoue T (2017) [The science of glaucoma surgery -Filtration surgery and the role of Cytokines]. Nippon Ganka Gakkai Zasshi 121(3):314–334 Japanese 30088704

[33] Lee J, Choi JA, Ju HH, Kim JE, Paik SY, Rao PV (2021) Role of MCP-1 and IL-8 in viral anterior uveitis, and contractility and fibrogenic activity of trabecular meshwork cells. Sci Rep 11(1):14950. 10.1038/s41598-021-94391-2 34294770 10.1038/s41598-021-94391-2 PMC8298573

[34] Suzuki K, Iwata D, Namba K, Hase K, Hiraoka M, Murata M, Kitaichi N, Foxton R, Ishida S (2023) Involvement of angiopoietin 2 and vascular endothelial growth factor in uveitis. PLoS ONE 18(11):e0294745. 10.1371/journal.pone.0294745 38015876 10.1371/journal.pone.0294745 PMC10683998

[35] Singh S, Anshita D, Ravichandiran V (2021) MCP-1: Function, regulation, and involvement in disease. Int Immunopharmacol. 101(Pt B):107598. 10.1016/j.intimp.2021.107598 10.1016/j.intimp.2021.107598 PMC8135227 34233864

